# Preparation of Graphene Oxide/Cellulose Composites with Microcrystalline Cellulose Acid Hydrolysis Using the Waste Acids Generated by the Hummers Method of Graphene Oxide Synthesis

**DOI:** 10.3390/polym13244453

**Published:** 2021-12-19

**Authors:** Yuanyuan Miao, Xiuya Wang, Yixing Liu, Zhenbo Liu, Wenshuai Chen

**Affiliations:** Key Laboratory of Bio-Based Material Science & Technology, Northeast Forestry University, Ministry of Education, Harbin 150040, China; miao.yuanyuan@nefu.edu.cn (Y.M.); wangxiuya2019@nefu.edu.cn (X.W.); tianliu@nefu.edu.cn (Y.L.)

**Keywords:** graphene oxide (GO), microcrystalline cellulose (MCC), composites, Hummers method, acidic mixture, acid hydrolysis

## Abstract

The Hummers method is the most commonly used method to prepare graphene oxide (GO). However, many waste acids remain in the raw reaction mixture after the completion of this reaction. The aim of this study was to reuse these waste acids efficiently. In this study, microcrystalline cellulose (MCC) was directly dissolved in the mixture after the high-temperature reaction of the Hummers method. The residual acid was used to hydrolyze MCC, and the graphene oxide/microcrystalline cellulose (GO/MCC) composites were prepared, while the acid was reused. The effects of MCC addition (0.5 g, 1.0 g, and 1.5 g in 20 mL) on the properties of the composites were discussed. The structure, composition, thermal stability, and hydrophobicity of GO/MCC composites were characterized and tested by SEM, XRD, FTIR, TG, and contact angle tests. The results showed that MCC could be acid hydrolyzed into micron and nano-scale cellulose by using the strong acidity of waste liquid after GO preparation, and it interacted with the prepared GO to form GO/MCC composites. When the addition amount of MCC was 1 g, the thermal stability of the composite was the highest due to the interaction between acid-hydrolyzed MCC and GO sheets. At the same time, the hydrophobic property of the GO/MCC composite is better than that of the GO film. The freeze-dried GO/MCC composites are more easily dispersed in water and have stronger stability.

## 1. Introduction

As an important oxygen-containing derivative of graphene, graphene oxide (GO) has the advantages of low production cost, large-scale production, and easy processing [1,2]. It is often used as a precursor for the preparation of reduced graphene oxide (RGO). In recent years, with the further research on GO, scientists have found that it also has the excellent performance of rich active oxygen-containing functional groups [3,4,5]. There are a large number of oxygen-containing functional groups such as carboxyl, hydroxyl, and epoxy groups on the surface and edge of GO [6,7,8]. These oxygen-containing groups can be used as catalytic activity centers. The covalent/non-covalent modification design can also be carried out according to the requirements of specific application fields [9]. So far, the functionalization of graphene oxide has made great progress. It has been applied to seawater desalination, drug delivery, oil–water separation, fixed catalysis, solar cells, energy storage, medical care, and other fields [10,11,12,13,14]. At present, relatively mature GO preparation methods are mainly divided into the Brodie method, Staudenmaier method, and Hummers method [15,16,17]. Their preparation process is to first mix graphite with inorganic strong protonic acid (such as concentrated sulfuric acid, fuming nitric acid, or their mixture) in a certain proportion, so that small molecules of strong acid are inserted into the graphite layer. Then it was oxidized by strong oxidants (such as KMnO_4_, KC10_4_, etc.). However, the Staudenmaier method and the Brodie method have the disadvantages of producing harmful gases such as ClO_2_ and NO_2_ during the reaction, consuming too much time, and consuming too much raw materials under long-term reaction conditions. Therefore, when GO is put into laboratory preparation and industrial production, the Hummers method is generally used as the preparation method of GO. The Hummers method has at least three important advantages over previous technologies. First, the reaction can be completed within a few hours. Secondly, replacing KClO_3_ with KMnO_4_ can improve the reaction safety and avoid the precipitation of explosive ClO_2_. Thirdly, NaNO_3_ was used to replace HNO_3_, which eliminated the formation of acid mist [18]. However, the Hummers method also has some room to be improved such as having too much acid residue that is difficult to remove, and the GO oxidation degree is not enough. Therefore, many researchers have made some improvements on the preparation of GO by the Hummers method. Marcano et al. have improved the Hummers method by increasing the amount of KMnO_4_ used, which increases the oxidation of GO [19]. Yu et al. prepared GO by partially replacing KMnO_4_ with K_2_FeO_4_ without NaNO_3_. This approach significantly reduces the consumption of reactants while maintaining sufficient GO oxidation [20]. However, since all the preparation processes need strong acid to treat graphite, acid residue is still a problem to be solved. So, we find it inevitable to use strong acids in the preparation of GO. So, the high-value utilization of residual acid, reducing the waste of raw materials and avoiding its impact on the environment, will become a new research starting point. This can also save the time consumed by centrifugation and dialysis during GO preparation.

Cellulose, a renewable, degradable, and environmentally friendly material, is abundant in nature. It is a natural polymer formed of dehydrated-D-glucose units linked by β(1→4) glycosidic bonds [21,22,23]. It is also one of the most common natural linear syndiotactic homopolymers [24,25]. Cellulose nanofibers show a high aspect ratio (up to 1 μm in length and 2–5 nm in thickness), a chemically modifiable surface, and a high elastic modulus resulting from their high crystallinity, and they are prepared by relatively low-cost production methods, all of which endow CNFs with great potential for the fabrication of numerous functional structures [26,27]. Inorganic acids are commonly used to hydrolyze cellulose-containing raw materials to prepare cellulose [28,29]. Microcrystalline cellulose (MCC) is a purified, partially depolymerized form of cellulose. It is worth noting that the numerous hydroxyl groups on the cellulose molecular chain may form hydrogen bonds with the oxygen-containing groups of GO [30,31]. In recent years, some researchers have made GO and cellulose into composites. The hydrogen-bonding connection between GO and MCC was used to prepare the composites with both the strong electrical properties, strong surface activity, and high thermal conductivity of GO and the green, renewable and excellent mechanical properties of MCC [32,33,34,35,36]. It is expected that this composite material can be applied in the adsorption, drug delivery, electrochemistry, and phase change energy-storage materials [37,38]. The methods used for composite preparation include solution blending methods, dissolution–combination methods (where ionic, urea-alkaline, or lithium bromide aqueous solutions are used to dissolve and combine with GO simultaneously), and cross-linking combination methods [39,40,41,42,43,44,45,46]. Although the research on GO/MCC composites has made some progress, there are still some problems to be solved. For example, it is difficult for natural cellulose to combine with GO directly, but the use of cellulose solvents (e.g., urea-alkaline or ionic liquids) or modified cellulose increases both composite production cost and the environmental burden.

In this study, from the perspective of conservation and environmental protection, a large amount of residual acid in the mixture after the reaction of the Hummers method was reused. After the preparation of GO, no washing and filtration treatment was performed on the used mixture, and MCC was directly added to make full use of the residual strong acid in the mixture to hydrolyze it, so that the GO/MCC composite was prepared while the agent was reused.

## 2. Materials and Methods

### 2.1. Materials and Chemicals

Ink powder is purchased from Tianjin Comiou Chemical Reagent Co., Ltd. (Tianjin, China); 98 wt % H_2_SO_4_ was purchased from Tianjin Kaitong Chemical Reagent Co., Ltd. (Tianjin, China). NaNO_3_ was purchased from Tianjin Kemiou Chemical Reagent Co., Ltd. (Tianjin, China). KMnO_4_ was purchased from Tianjin Kaitong Chemical Reagent Co., Ltd. (Tianjin, China). H_2_O_2_ was purchased from Tianjin Kaitong Chemical Reagent Co., Ltd. (Tianjin, China). MCC (diameter: 90 μm) was purchased from Shanghai Aladdin Biochemical Technology Co., Ltd. (Shanghai, China).

### 2.2. Graphene Oxide (GO) Preparation

GO was prepared according to the modified Hummers method [6], with a slight adjustment to the amount of water added during the high-temperature reaction. In this portion of the reaction, 46 mL of concentrated H_2_SO_4_ (98 wt.%) was added to a beaker and cooled in an ice water bath to about 4 °C. Next, 2.0 g of graphite powder and 1.0 g of NaNO_3_ were added to the beaker, which was followed by the slow addition of 6.0 g KMnO_4_. The reaction was allowed to progress at <10 °C for 90 min; then, the ice water bath was replaced with a warm water bath. The reaction proceeded for 30 min at 35 °C. After the 30-minute reaction time, 46 mL of deionized water was added dropwise to the reaction solution, and the temperature of the reaction was maintained at 95 °C for at least 15 min. The concentration of sulfuric acid in the final mixture was about 60 wt.%. An appropriate amount of 30 wt.% H_2_O_2_ was added to the raw reaction mixture until no more visible bubbles were produced, and the mixture was allowed to stand.

### 2.3. Preparation of the GO/MCC Composite

After the Hummers acidic mixture was prepared (as described above), it was stirred evenly. Four 20 mL aliquots were removed. One aliquot, left untreated, was used as the control. Three MCC samples (0.5 g, 1 g, and 1.5 g) were each dissolved in one aliquot of the Hummers raw reaction mixture at 45 °C for 1 h. Then, all samples were sonicated for 30 min in an ice water bath, which was followed by dialysis and washing until the solution was neutral. These samples are herein referred to as “GO/C-0.5 g,” “GO/C-1 g,” and “GO/C-1.5 g.” The control is referred to as “GO”. All samples were freeze-dried and then dispersed in deionized water to generate 2 mg mL^−1^ dispersions, and 12 mL dispersions of each sample were filtered with vacuum filtration into a thin film for contact angle measurement.

### 2.4. Characterization

Scanning electron microscope (SEM) images were obtained using a QUANTA200 (FEI, Eindhoven, The Netherlands) instrument. Fourier transform infrared spectroscopy (FTIR) is performed on a Spectrum 400 (PE Company, New York, NY, USA) instrument with a scanning range of 550–4000 cm^−1^. X-ray diffraction (XRD) analysis was performed using XRD-6100 (Shimadzu Corporation, Kyoto, Japan) in the scanning range of 5–60° and the scanning speed of 5°/min. Raman spectroscopy (RDS) was measured by Renishaw in Via instrument (Renisho, Wotton-under-Edge, UK). A thermogravimetric analysis (TG) diagram was obtained by high-precision thermogravimetric analyzer (/TG209F1, Niche, Berlin, Germany), and the test temperature range was 5–790 °C. A contact angle test was obtained with a Dataphysics-OCA20 instrument (Dataphysics, Filderstadt, Germany).

## 3. Results

### 3.1. FTIR

**Figure 1:**
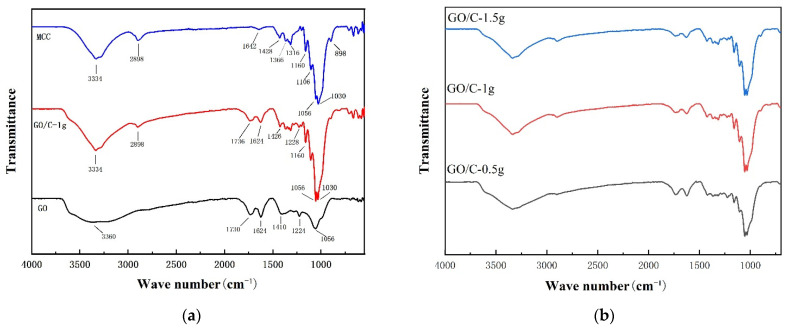
The FTIR spectra of (**a**) microcrystalline cellulose (MCC), graphene oxide (GO), GO plus 0.5 g MCC composite (GO/C-1 g); (**b**) GO plus 0.5 g MCC composite (GO/C-0.5 g), GO plus 1 g MCC composite (GO/C-1 g), and GO plus 1.5 g MCC composite (GO/C-1.5 g).

Obvious characteristic peaks of GO can be seen from FTIR in Figure 1a, including C=O (1730 cm^−1^), the conjugated structure C=C (1624 cm^−1^), the carboxyl group C–O (1410 cm^−1^), the epoxy structures C–O (1224 cm^−1^) and C–O (1056 cm^−1^), and the strong broad peak at around 3340 cm^−1^ [47,48]. The strong broad peak near 3340 cm^−1^ is the stretching vibration of –OH [49]. The FTIR spectrum of the MCC was clearly consistent with the spectral characteristics of cellulose, showing the characteristic stretching vibrations of –OH at 3334 cm^−1^ and –CH_2_ at 2898 cm^−1^. The peaks at 1200–1500 cm^−1^ were characteristic of the bending and rocking vibrations of –CH, –CH_2_, and –OH, while the multiplet at 800–1200 cm^−1^ reflected the skeleton vibration of the diether structure C–O–C–O–C. Although the FTIR spectra of the GO/C composites were similar to that of MCC, and the main absorption peaks of the composites were identical to those of cellulose, the interactions between GO and MCC in the composites were still reflected in the spectra. Compared with the FTIR spectra of GO or MCC, the O–H and C–H peaks of GO/C composites were relatively shifted. This indicates that many O–H and C–H in the composites form hydrogen bonds, and GO is effectively combined with MCC. It is worth noting that for the GO/nanocrystalline cellulose (MCC) composites prepared using the urea-alkaline system, the characteristic –COOH peak (≈1730 cm^−1^) completely disappeared due to acid–base neutralization [50]. However, when the sample was prepared in the acidic solution, this peak (at 1735 cm^−1^) was observed.

The FTIR spectra of the samples prepared with different amounts of added MCC were similar without significant shifts in the positions of the absorption peaks (Figure 1b). Therefore, a different amount of added MCC did not affect the types of functional groups in the composite sample.

### 3.2. XRD

**Figure 2:**
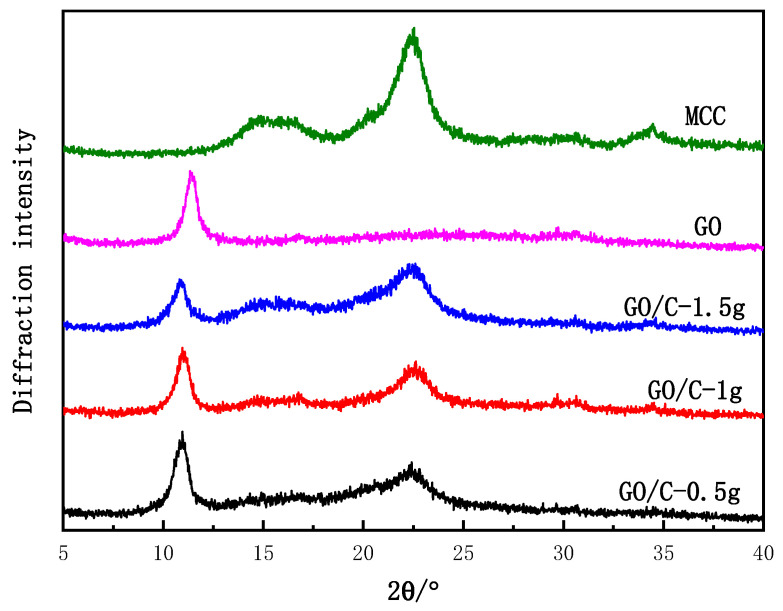
The XRD spectra of microcrystalline cellulose (MCC), graphene oxide (GO), GO plus 0.5 g MCC composite (GO/C-0.5 g), GO plus 1 g MCC composite (GO/C-1 g), and GO plus 1.5 g MCC composite (GO/C-1.5 g).

Pure GO had a characteristically strong XRD peak at 2θ = 11.4° (Figure 2), corresponding to the (001) crystal plane of GO. In addition, according to the Bragg equation, the layer spacing of GO was estimated at 0.77 nm, which is similar to results reported in the literature [51]. The MCC XRD peaks appeared at 2θ = 14.8°, 16.5°, 22.5°, and 34.5°, corresponding to the crystal planes 101, 10$\bar{1}$, 002, and 040, respectively. This result indicated that MCC had a cellulose I-type crystal structure [52,53]. The XRD pattern of GO/C-1 g was similar to those of GO and MCC: a strong diffraction peak was observed at 10.9°, which was slightly left-shifted from the characteristic GO diffraction peak, and at 22.5°, which was consistent with the characteristic peak position of the 002 crystal plane of cellulose I. Furthermore, two smaller diffraction peaks were found at 2θ = 14.9° and 16.7°, corresponding to the 101 and 10$\bar{1}$ crystal planes of cellulose I, respectively. The amount of MCC added had almost no impact on the position of the characteristic peaks of the composite (Figure 2). As the amount of added MCC increased, the intensity of the diffraction peak of the composite at around 2θ = 11° decreased, but the characteristic peak intensity of cellulose was somewhat increased. This result indicated that as the amount of added MCC increased, the interactions between the acid-hydrolyzed cellulose and the GO sheet layer also increased, disrupting the order of the GO sheet and reducing the intensity of the characteristic peak. As the added MCC increased, the proportion of cellulose in the composite material increased concomitantly, increasing the intensity of the characteristic cellulose peak (Figure 2). A hydrogen bond was formed between GO and MCC, which also confirmed the results of FTIR.

### 3.3. RDS

**Figure 3:**
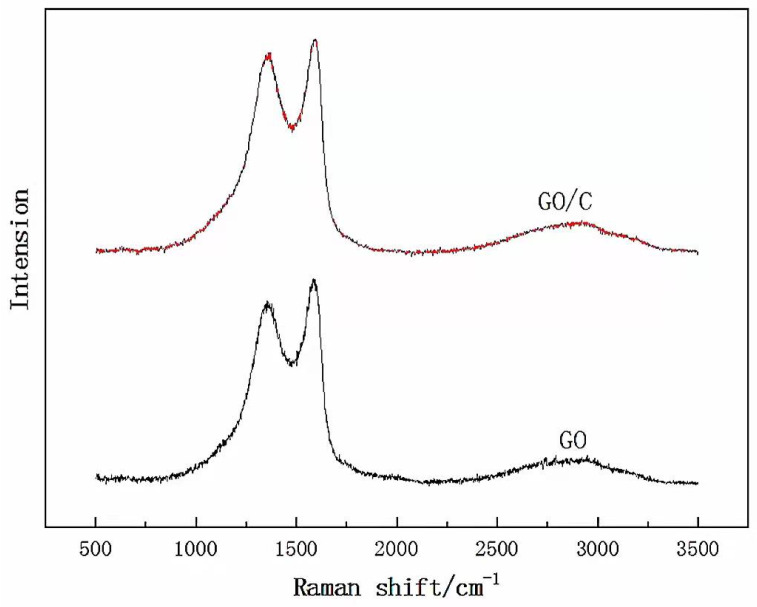
The Raman spectra of graphene oxide (GO) and GO plus 1 g MCC composite (GO/C-1 g).

Figure 3 shows the Raman spectra of GO and GO plus 1 g MCC composite (GO/C-1 g). The two curves are similar, both showing a D band (1371 cm^−1^, which corresponds to the disordered structure of GO sheets) and a G band (1586 cm^−1^, which represents the first-order scattering of the E2g vibrational mode). The small difference between the two spectra may be due to the fact that Raman signals are very sensitive to conjugate structures, and the complex GO/cellulose still contains a large number of conjugate π bond structure. So, the GO/C spectrum is extremely similar to the pure GO one without an obvious characteristic peak of cellulose [54]. There is a wide peak with low intensity between 2500 and 3250 cm^−1^, which is caused by the overlapping of the 2D peak, D + G peak, and 2D’ peak, which is consistent with previous studies [55]. As shown in Table 1, according to determination of the intensity ratio of the D peak to the G peak, ID/IG, we found that the ID/IG value of the GO/cellulose composite was 1.62, which was slightly larger than the ID/IG value of GO (1.57), which proved that there was an interaction between MCC and GO, which increased the ID/IG value of the GO/cellulose composite.

### 3.4. TG

**Figure 4:**
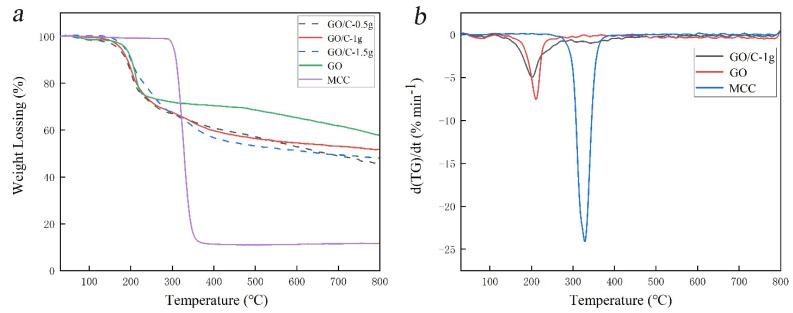
The TG and derivative TG (DTG) curves of the samples: (**a**) TG curve; (**b**) DTG curve.

It can be seen from the TG/DTG curves in Figure 4 that the thermal decomposition of MCC, GO, and composites is mainly completed in one stage. The thermal weight loss value is shown in Table 2. It can be seen from Table 2 that the *T*_0_ values of the three composites are smaller than those of MCC and GO, which is caused by the introduction of sulfonic acid groups during acid hydrolysis [56,57,58]. The TG/DTG curves of the composites are obviously different from those of MCC and GO. However, the thermal decomposition of MCC occurred in one stage, indicating that the acid hydrolysis of MCC occurred during the preparation process, and the composite was formed by the interaction between MCC and GO sheets. This result was consistent with the conclusions of XRD and FTIR. Due to the poorer thermal conductivity of cellulose than GO, the interlacing of cellulose and GO sheets in GO/C-1 g hindered the heat transfer. This makes the thermal decomposition temperature range of GO/C-1 g significantly wider than that of GO, and *T_f_* is higher than that of GO. With the increase in MCC content, the more sulfonic groups introduced into the composites during acid hydrolysis, and the lower the *T*_0_ value. When the addition amounts of MCC were 0.5 g and 1 g, the interaction between acid-hydrolyzed cellulose and the GO sheet could improve the thermal stability of the composites, and the carbon residue of the composites was also higher. However, as the addition of MCC continues to increase, the more insufficient the acid hydrolysis reaction is, the more large-scale MCC remains. The carbon residue of MCC is relatively low, which makes the carbon residue of GO/C-1.5 g lower than GO/C-1 g. Therefore, when the MCC content is 1 g, the thermal stability of the composite reaches the maximum.

### 3.5. Contact Angle Test the Initials

**Figure 5:**
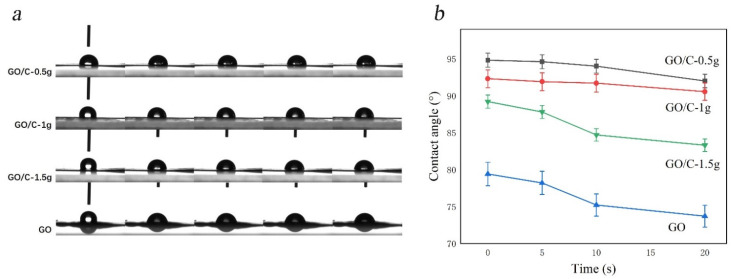
Alterations in water contact angles across all samples. (**a**) The contact angle test process of the samples (left to right: 1 s, 3 s, 5 s, 10 s, 20 s); (**b**) Contact angle image of hydrophobic of samples.3.5. SEM.

The distilled water contact angles of the GO, GO/C-0.5 g, GO/C-1 g, and GO/C-1.5 g sample films were tested. Alterations in contact angle were detected within 20 s (Figure 5). At all time points, the contact angles measured for GO/C-0.5 g, GO/C-1 g, and GO/C-1.5 g were greater than that of GO (Figure 5a), which was probably because of hydrogen bonding between cellulose and GO, and the resultant decrease in the number of oxygen-containing groups on the GO sheet. Thus, the hydrophobicity levels of the composites were higher than that of GO. As shown in Figure 5b, the hydrophobicity of the composites decreased with the addition of MCC. The hydrophobicity of GO/C-0.5 g was higher than that of GO/C-1 g. However, there was no significant difference between them. The water contact angles of GO/C-0.5 g and GO/C-1 g were 91.85° and 90.55° at 20 s, respectively. However, with the addition of MCC, the water contact angle difference is gradually obvious. The gap between GO/C-1 g and GO/C-1.5 g is larger than that between GO/C-0.5 g and GO/C-1 g. This is because the hydrophobicity of cellulose is poor. With the continuous addition of MCC, the hydrophobicity of the composite decreases. In addition, when the MCC content continues to increase, the incomplete acid hydrolysis of MCC may also affect the hydrophobicity of the composites. Overall, the composites are more hydrophobic than GO. The hydrophobicity of GO/C-0.5 g and GO/C-1 g in the composites is more excellent.

**Figure 6:**
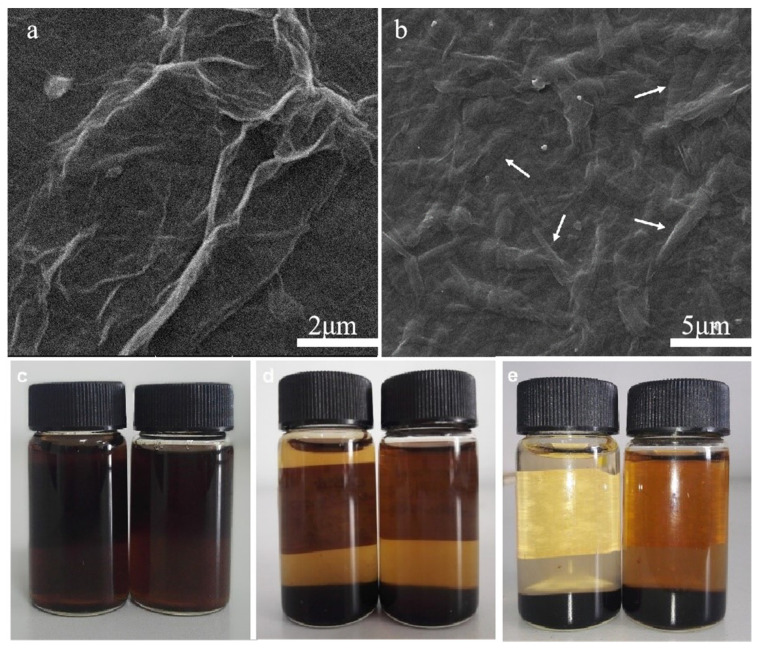
Microscopic and macroscopic morphologies of the samples during sample preparation. (**a**) SEM image of graphene oxide (GO); (**b**) SEM image of GO plus 1 g MCC composite (GO/C-1 g); (**c**–**e**) the photos of GO (left) freeze-dried powder and GO/C-1 g (right) freeze-dried powder dispersed in water on the 1st, 6th, and 78th days, respectively.

After drying of the GO aqueous dispersion, environmental scanning electron microscopy (SEM) images were produced (Figure 6a). Folded GO sheets were clear in the SEM images, indicating that the addition of water to the high-temperature reaction did not affect the preparation of the GO sheets. In the SEM image of the GO/C-1 g sample, it was clear that MCC had been acid-hydrolyzed into short rod-shaped cellulose molecules with non-uniform sizes (Figure 6b). The diameter of all cellulose rods was <1 μm, with a length of 2–6 μm; the cellulose rods were covered by GO sheets. This indicates that MCC in GO/C-1 g sample has been mostly or completely hydrolyzed. This also provided a microscopic explanation for the hydrophobicity similarity of GO/C-0.5 g and GO/C-1 g samples.

Figure 6c–e show photographs of GO (left) freeze-dried powder and GO/C-1 g (right) freeze-dried powder dispersed in water at a concentration of 2 mg/mL on days 1, 6, and 78, respectively. As shown in Figure 6c, the mixture of the two samples showed a relatively stable state in the initial three days after dispersion. There is no obvious precipitation at the bottom of the sample bottle. As shown in Figure 6d, with the passage of time, partial precipitation appeared at the bottom of the two sample bottles on the sixth day, and the upper liquid gradually became clear. As shown in Figure 6e, the color of the upper liquid of GO/C-1 g (right) composite is obviously deeper than that of GO (left) after 78 days of storage. This shows that the stability of the GO/C-1 g composite dispersion is stronger than that of GO. This is because the acid hydrolysis cellulose after GO preparation was used in the experiment, and there were micron and nano-scale cellulose in GO/C composites. The depolymerization of cellulose macromolecular chains and the breaking of hydrogen bonds are the main factors leading to the depolymerization of cellulose in sulfuric acid [59]. H_2_SO_4_ aqueous solution with adequate concentrations is able to break the hydrogen bonds, because H^+^ favors the attack on oxygen atoms of cellulose hydroxyl groups, and bisulfate and sulfate anions prefer to attract hydrogen atoms of cellulose hydroxyl groups [60]. After acid hydrolysis, a small amount of negative charges are introduced on the surface of nanocellulose, and the mutual exclusion of negative charges makes these composites have a strong dispersion ability in water [61,62]. In addition, one-dimensional cellulose was interspersed between two-dimensional GO sheets. There was a certain steric effect between the two, which hindered the agglomeration between GO sheets and cellulose, respectively, which was also conducive to the stable dispersion of GO/C in the solution [63]. Thus, the stability of GO/C composites in water is higher than that of GO.

## 4. Conclusions

In this study, the mixed acid solution of GO prepared by the Hummers method was used to directly dissolve MCC, and GO/MCC composites were prepared after washing, dialysis, and ultrasonic treatment. The XRD, FTIR, TG, and SEM test data of the samples show that this method is feasible. GO and MCC are connected by hydrogen bonding. The pyrolysis range of the composite is wider than that of GO, and its hydrophobicity to distilled water is also better than that of the GO film. The composite was easily dispersed in water again after freeze drying, and the stability of the solution was slightly stronger than that of GO. When the addition of MCC is 1 g, the composite has better thermal stability and hydrophobic properties. Compared with the preparation methods such as the solution blending method and dissolution–composite method, the preparation in this study realized the secondary utilization of acid, saved the preparation cost, and reduced the impact on the environment to a certain extent. It also provides a new idea for the high-value preparation of GO composites. At the same time, this GO/MCC composite with good thermal stability, hydrophobicity, and long-term storage has a certain potential application value in hydrophobic materials.

## Figures and Tables

**Table 1:** The analysis result of Raman spectra.

	Peak Position		Peak Intensity		Half-Height Width		Integral Area		R
	D	G	D	G	D	G	D	G	Value
GO	1371.05	1585.88	959.16	1002.08	282.21	107.28	430,892.93	275,297.52	1.57
GO/C-1 g	1368.72	1588.97	1041.26	1057.33	288.41	103.90	476,112.35	294,397.92	1.62

**Table 2:** Thermal analysis data for MCC, GO and GO/C.

Sample Code	^1^*T*_0_/°C	^2^*T*_max_/°C	^3^*T_f_*/°C	^4^*M*_800_/%
MCC	307	328.8	345.0	11.66
GO	194	211.0	223.1	57.79
GO/C-0.5 g	177	201.7	233.6	45.53
GO/C-1 g	175	201.6	253.7	51.84
GO/C-1.5 g	169	204.0	297.2	48.12

^1^*T*_0_—the extrapolated onset temperature of the thermal decomposition peak (also the extrapolated onset temperature of the derivative TG (DTG) curve). ^2^*T*_max_—the temperature at the maximum weight loss rate. ^3^*T_f_*—the extrapolated final temperature of the DTG curve ^4^*M*_800_—the residual carbon content at 800 °C.

## Data Availability

The data that support the findings of this study are available from the corresponding author upon reasonable request.
